# Prevalence, indications, and outcomes of operative vaginal deliveries among mothers who gave birth in Ethiopia: A systematic review and meta-analysis

**DOI:** 10.3389/fgwh.2022.948288

**Published:** 2022-09-22

**Authors:** Bikila Balis, Adera Debella, Indeshaw Ketema, Bajrond Eshetu, Ebisa Zerihun, Alemayehu Deressa Wayesa, Sisay Habte, Adisu Alemu, Habtamu Bekele

**Affiliations:** ^1^School of Nursing and Midwifery, College of Health and Medical Sciences, Haramaya University, Harar, Ethiopia; ^2^Department of Nursing, College of Health Science, Oda Bultum University, Chiro, Ethiopia; ^3^School of Public Health, College of Health and Medical Sciences, Haramaya University, Harar, Ethiopia

**Keywords:** vacuum extractor, operative vaginal deliveries, instrumental deliveries, systematic review, meta-analysis, obstetrics forceps

## Abstract

**Background:**

Operative vaginal deliveries represent an alternative to address problems during the second stage of labor. Clinicians have access to two different instruments obstetrics forceps and vacuum which should be conducted with indication. Understanding the pooled prevalence of operative vaginal deliveries, its indications, and outcomes would help in adopting suitable measures to reduce operative vaginal deliveries-related maternal and neonatal complications. Therefore, this systematic review and meta-analysis aimed to determine the prevalence, indications, and outcomes of operative vaginal deliveries among mothers who gave birth in Ethiopia.

**Methods:**

A literature search was done through databases such as PubMed, SCOPUS, Web of Sciences, CAB Abstract, and CINHAL (EBSCO) to search studies that have been conducted in Ethiopia. Relevant sources were consulted to retrieve unpublished studies. Original observational studies that reported the prevalence, indication and outcomes of operative vaginal deliveries conducted in the English language were identified and screened. Studies were independently assessed for inclusion, data extraction, and risk of bias.

**Results:**

Twelve studies were reviewed. The overall pooled prevalence of operative vaginal delivery among mothers who gave birth in Ethiopia was 10% (95% CI: 8 to 13) with *I*^2^ = 98.82% and a *p*-value ≤ 0.001. Fetal distress, prolonged labor, and maternal exhaustion were the most common feto-maternal indications of OVDs whereas; neonatal death, poor Apgar score, admission to neonatal intensive care unit, perianal tear, and postpartum hemorrhage were complications that occur following the operative vaginal deliveries in Ethiopia.

**Conclusion:**

This systematic review and meta-analysis showed one out of 10 mothers undergo operative vaginal deliveries. Almost all feto-maternal complications that arise following operative vaginal deliveries were preventable. Thus, concerned stakeholders should encourage quality OVDs practice by avoiding unnecessary indications and scaling up the skill of health professionals through special training.

**Systematic review registration:**

http://www.crd.york.ac.uk/PROSPERO/, identifier CRD42022311432.

## Introduction

Despite increased cesarean-section deliveries over the past few decades, operative vaginal deliveries remain an important component of modern labor management, accounting for 3.3% of all deliveries in 2013 (1). Operative vaginal deliveries (OVDs) refer to obstetrics forceps and/or vacuum-assisted delivery to expedite the second stage of labor and help to decrease the primary cesarean-section rate (2). This performed by trained care providers to minimize maternal and neonatal complications (1). Even though operative vaginal deliveries have been used in the past centuries as an alternative mode of delivery, the recent trends have demonstrated that particularly forceps use has declined as cesarean delivery rate is increasing (3–5). In Ethiopia, prevalence of operative vaginal delivery among mothers who gave birth with a range of 2.10–27.90% (6, 7).

Most of the time operative vaginal deliveries are indicated for shortening second stage of labor in case of maternal exhaustion, non-reassuring fetal heart rate tracing, or women with conditions that contraindicate pushing during labor. Furthermore, OVDs are performed only with prerequisites such as an engaged fetal head, fully dilated cervix, ruptured membrane, identified fetal head position, and an intact uterus (8, 9).

Although operative vaginal deliveries involves a risk for maternal and neonatal complications (10, 11), many women prefer OVDs than caesarian section due to longer recovery and downstream health effects associated with cesarean section (1, 12). Both forceps and vacuum have the potential to cause maternal and neonatal injury; however, the incidence of maternal injury is more with the forceps than with vacuum. Unless it is performed by trained and experienced health workers, OVDs can cause injury to pelvic floor and compromise's urinary, genital and gastro-intestinal system. According to Biru et al. perianal laceration is the most common maternal complication (13) whereas a low APGAR score is the most common neonatal complication (14). So that in order to minimize the complications, OVDs are recommended to be performed from either a low or outlet station (1, 8).

Despite few studies conducted on operative vaginal deliveries among women who gave birth in Ethiopia, there is lack of data's which represent national-level or cumulative evidence to show the exact level and the wider impact of OVDs. The prevalence of operative vaginal deliveries in Ethiopia setting is investigated in disjointed ways. Hence, there is a need for an inclusive study that can aggregate previous studies to make it an input for decision makings. Therefore, this systematic review and meta-analysis were conducted to generate evidence-based, nationally reliable pooled evidence on prevalence of operative vaginal deliveries, their indication, and feto-maternal outcomes among women who gave birth in Ethiopia.

For this review, the following operational definition was used; Operative vaginal deliveries: refer to methods of shortening the second stage of labor using obstetrics forceps and/or vacuum extractors for maternal and fetal purposes.

## Methods

### Study protocol and registration

This systematic review and meta-analysis were conducted to determine the pooled prevalence, indications, and outcomes of OVDs among mothers who gave birth in Ethiopia. The findings of this review were reported following the Preferred Reporting Items for Systematic Review and Meta-Analysis (PRISMA) checklist (15). We also employed the Meta-analysis of observational Studies in Epidemiology (MOOSE) guidelines to conduct the meta-analysis and to report the results (16). The review was registered by the International prospective register of systematic reviews with identification CRD42022311432.

### Eligibility criteria

This review included all cross-sectional study designs conducted in Ethiopia. Moreover, all observational studies with the primary objective to determine the prevalence, indications, and outcomes of OVDs among mothers who gave birth in Ethiopia were considered for inclusion. Similarly, unpublished and published studies written in English were retrieved and included in the review process irrespective of their publication year. However, we excluded the studies that did not reported prevalence OVDs, their indications and outcomes, studies that failed to fulfill the quality criteria, experimental studies, reviews, commentaries, editorials, and case series/reports.

### Search strategy

Electronic databases such as PubMed, SCOPUS, Web of Sciences, CAB Abstract, and CINHAL (EBSCO) were searched for studies that have been conducted in Ethiopia. Also, relevant sources such as Google search engine, Google scholar, and WHO websites were searched. In addition, experts in the field were consulted to retrieve unpublished studies.

The search strings have emerged from the following keywords (operative vaginal delivery, instrumental delivery, forceps delivery, Vacuum delivery, indications of operative vaginal delivery, outcomes of operative vagina delivery, Ethiopia). Depending on the specific requirement of the database, the search strings were modified, and relevant studies using search strings were identified. The combinations of Boolean logic operators (AND, OR, NOT), free keywords, and MeSH (medical sub-headings) will be extensively used in the search process. For instance, PubMed search: ((((“forceps delivery”[All Fields]) OR (Vacuum delivery)) OR (“operative delivery”[All Fields])) OR (“instrumental delivery”[All Fields])) AND (“ethiopia”[All Fields]). The reference lists of relevant studies were also reviewed for sources that may have been missed in the database search. The search strategy developed for the selected database was attached (Supplementary material).

### Study selection

Initially, all the articles obtained from the selected databases were exported to the EndNote X8 version library and exact duplicates were removed. Then, the EndNote library was shared between two reviewers (BB and BE) who independently screened articles by title and abstract. Any disagreements between the reviewers were solved through discussion or through asking others reviewers (HB, AD, IK, and EZ) if consensus could not be reached. After reaching a consensus, the full-text review was performed by the three reviewers (BB, IK, and BE) independently. The overall study selection process is presented using the PRISMA statement flow diagram.

### Data extraction

Two reviewers (BB and BE) independently extracted the data from the full text of the retained articles. A pre-defined Microsoft excels 2010 format was used to extract the data from selected studies under the following heading: author, publication year, region, setting, study design, sample size, study subject and primary outcomes of interest. The accuracy of the data extraction was verified by comparing the results of the independently extracted data.

### Quality and risk of bias assessment

The quality of retained articles was appraised independently by the four reviewers (BB, BE, HB, and AD) using an adapted version of the Newcastle–Ottawa Scale (17), since all of the articles that fulfilled the inclusion criteria were conducted with cross-sectional designs. The mean score of the authors' assessment was taken for a final decision. The differences in the inclusion of the studies were resolved by consensus. The included studies were evaluated against each indicator of the tool and categorized as high, moderate, and low quality. A high-quality score above 80%, moderate-quality between 60 and 80%, and low-quality below 60%. Articles with a score ≥ 60% were included. This critical appraisal was conducted to assess the internal validity (systematic error) and external validity (generalizability) of the studies and to reduce the risk of biases. Quality scores for each article are presented in Supplementary material.

### Outcome measures and statistical analysis

The primary outcome variable of this review was the prevalence of OVDs among mothers who gave birth and was measured based on the proportion of mothers who gave birth either by obstetrics forceps or vacuum extractor. The prevalence of OVDs among mothers who gave birth reported in different studies was presented by pooling the prevalence of OVDs among mothers who gave birth reported in the included articles. To take the study-specific true effects across the included studies into consideration, the random effect meta-analysis model was employed. A random-effects model for the reported proportion was used to present the pooled prevalence of OVDs among mothers who gave birth.

Data synthesis and statistical analysis were conducted using STATA 14 software. Forest plots were used to show the prevalence of OVDs, their indications, and outcomes among mothers who gave birth in Ethiopia. Subgroup analyses were also conducted by different study characteristics such as regions, sample size, and publication year.

The investigators checked for potential publication bias through visual inspection of a funnel plot, and Egger's Regression Test. Also, the *P*-value of < 0.05 for *I*^2^ statistics was used to determine the presence of heterogeneity. Similarly, low, moderate, and marked heterogeneity was assigned to I^2^ test statistics results of 25, 50, and 75%, respectively. The results of the review were reported according to the PRISMA guidelines. The findings of the included studies were presented using a narrative synthesis.

## Results

### Description of the studies

**Figure 1:**
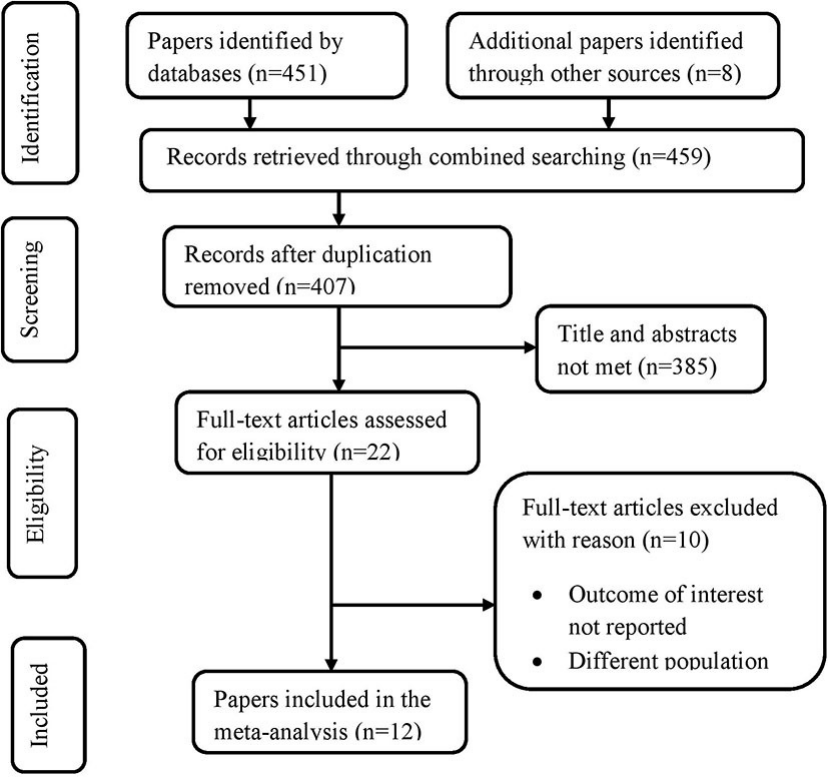
PRISMA statement presentation for systematic review and meta-analysis of OVDs, its indication, and outcomes among mothers who give birth in Ethiopia, 2022. n, number of studies included at each level.

Four hundred fifty-nine (459) papers were identified by electronic databases and other relevant sources. All papers were exported to the endnote library and duplicates (52 papers) were removed. Also, a total of 385 unrelated papers were excluded after titles and abstracts were reviewed. Then, twenty-two (18) papers were retrieved for detailed examination, and after a full-text review of those papers, ten (10) papers were excluded due to different populations and outcomes of interest not reported. Finally, full texts of the remaining twelve (12) papers were selected for the methodological quality assessment using JBI critical appraisal tools. All appraised papers met the inclusion criteria and were included in the review. All studies that examined the prevalence, indications, and outcomes of operative vagina deliveries among mothers who gave birth were included in the final review. Below is the PRISMA presentation for the selection of studies (Figure 1).

### Characteristics of included studies

Twelve cross-sectional primary studies with a sample size of 3,245 were used to determine the pooled prevalence of OVDs among mothers who gave birth in Ethiopia. The prevalence of OVDs included in the reviews ranged from 2.1 to 27.2%. Of those included studies, six were conducted in SNNPR, three in Amhara, and the left three studies were found in other places in Ethiopia (Table 1).

**Table 1:** General characteristics of studies included in the systematic reviews and meta-analysis among mother who gave birth in Ethiopia, 2022.

**References**	**Publication year**	**Region**	**Setting**	**Study design**	**Study subject**	**Sample size**	**Prevalence**	**Event**
Abdo et al. (6)	2020	SNNPR	IB	CS	Mothers who gave birth	4,004	2.10%	85
Biru et al. (13)	2017	SNNPR	IB	CS	Mothers who gave birth	14,688	5.72%	841
Hubena et al. (19)	2018	Oromia	IB	CS	Mothers who gave birth	2,348	10.30%	242
Abebaw et al. (20)	2021	Addis Ababa	IB	CS	Mothers who gave birth	12,995	11.90%	1,547
Asratie et al. (21)	2021	Amhara	IB	CS	Mothers who gave birth	548	7.10%	39
Tamirat et al. (22)	2022	SNNPR	IB	CS	Mothers who gave birth	377	13.80%	52
Shaka et al. (14)	2019	SNNPR	IB	CS	Mothers who gave birth	2,613	8.66%	216
Woretaw et al. (23)	2021	Amhara	IB	CS	Mothers who gave birth	410	15.60%	64
Beyene et al. (24)	2020	Amhara	IB	CS	Mothers who gave birth	411	10.90%	45
Yemaneh et al. (7)	2017	Tigray	IB	CS	Mothers who gave birth	338	27.20%	92
Bago et al. (25)	2018	SNNPR	IB	CS	Mothers who gave birth	414	3.00%	13
Shiferaw et al. (26)	2017	SNNPR	IB	CS	Mothers who gave birth	80	11.25%	9

IB, Institutional based; SNNPR, Southern Nation, Nationality and People Region; CS, cross-sectional.

### The pooled prevalence of operative vaginal deliveries

**Figure 2:**
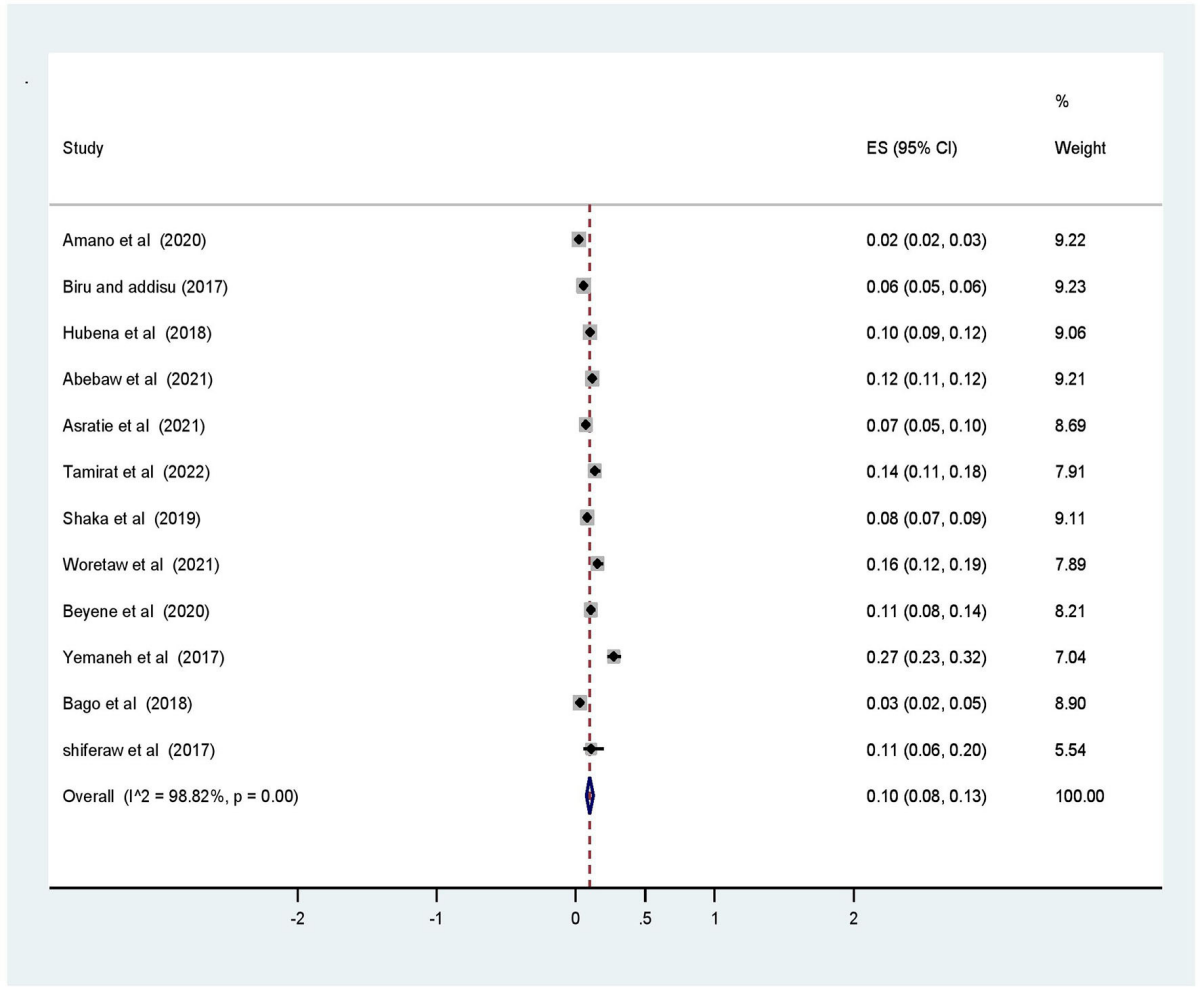
Forest plot of the pooled prevalence of operative vaginal deliveries among mothers who gave birth in Ethiopia, 2022.

The overall pooled prevalence of operative vaginal deliveries among mothers who gave birth in Ethiopia was 10% (95% CI (8.0, 13.0) with *I*^2^ = 98.82% and a *p*-value ≤ 0.001 (Figure 2). However, we conducted analysis for those studies who separately reported vacuum delivery and forceps. Thus far, the pooled prevalence of forceps delivery and vacuum delivery was 7.0% (95% CI (4.0, 10.0) with *I*^2^ = 98.76%, a *p*-value ≤ 0.001 and 4.0% (95% CI (3.0, 6.0) with *I*^2^ = 94.43%, *p*-value ≤ 0.001, respectively.

### Publication bias

**Figure 3:**
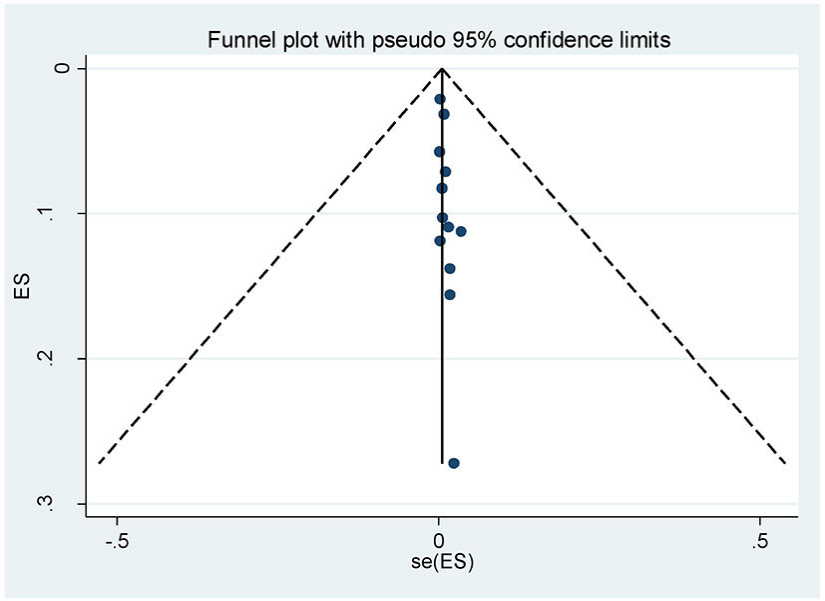
Funnel plot for pooled prevalence of operative vaginal deliveries among mothers in Ethiopia, 2022.

To observe publication bias, the Egger regression test revealed no evidence of publication bias among the included studies (*p* = 0.197) in addition a visual inspection of the funnel plot was done and presented in the figure below (Figure 3).

### Meta-regression to check the heterogeneity for prevalence of operative vaginal deliveries

A meta-regression analysis was conducted since there was statistically significant heterogeneity, with I-square test statistics < 0.05. The purpose of the analysis was to identify the source of heterogeneity so that the correct interpretation of the findings is made. However, the meta-regression analysis found no significant variable which can explain the heterogeneity. There was no statistically significant study level covariate: sample size and publication year of included studies. Therefore, the heterogeneity can be explained by other factors not included in this review (Table 2).

**Table 2:** Meta-regression analysis to check heterogeneity on operative vaginal deliveries among mothers who gave birth in Ethiopia, 2022.

**Variables**	**Coefficients**	**SE**	***P***	**[95% Conf. Interval]**
Publication year	−0.0029176	0.0118161	0.811	−0.0296474, 0.0237034
Sample size	−3.1306	4.1006	0.465	−0.0000124, 6.14006

### Subgroup analysis

**Figure 4:**
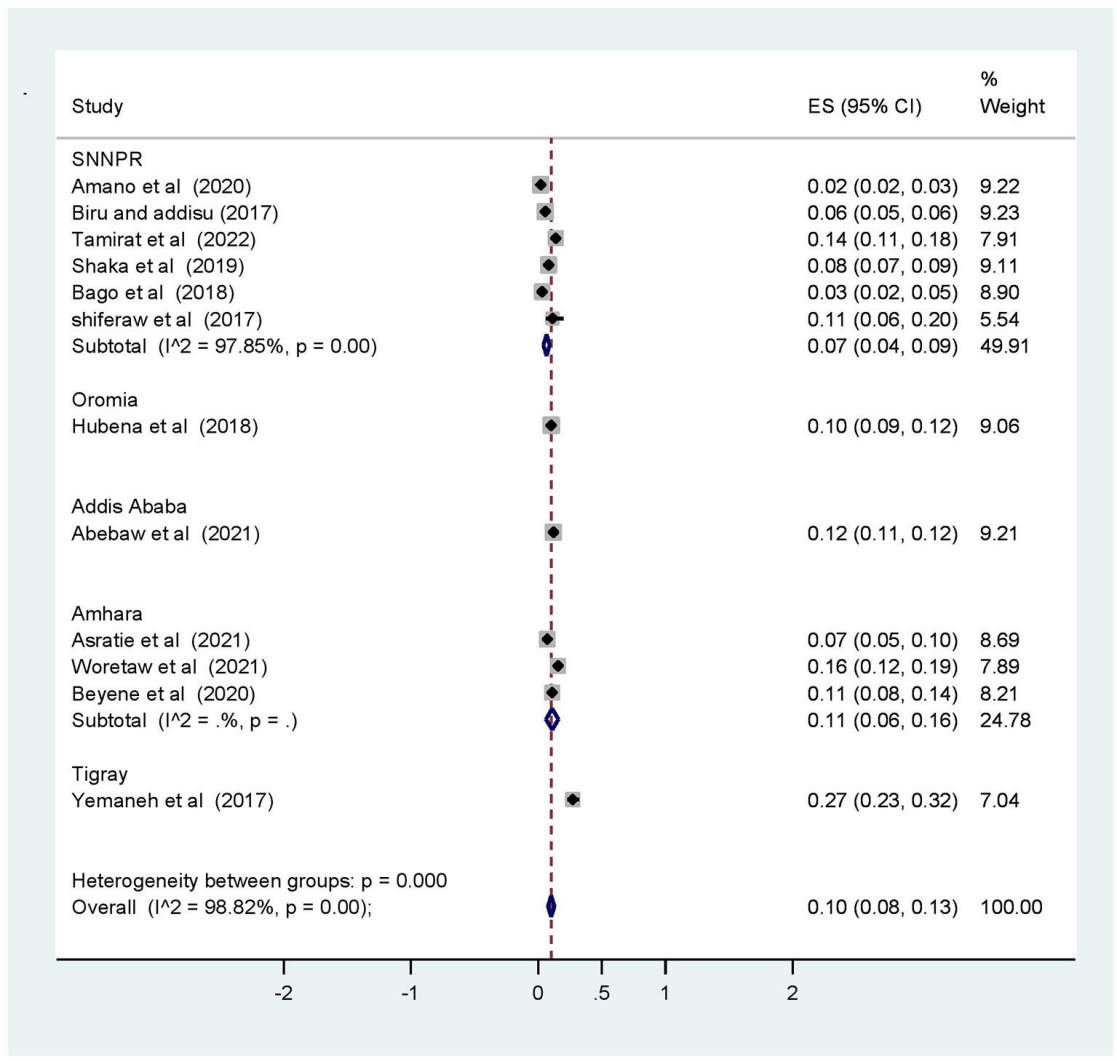
Subgroup analysis of prevalence of operative vaginal deliveries based on region (area) among mothers who gave birth in Ethiopia, 2022.

**Figure 5:**
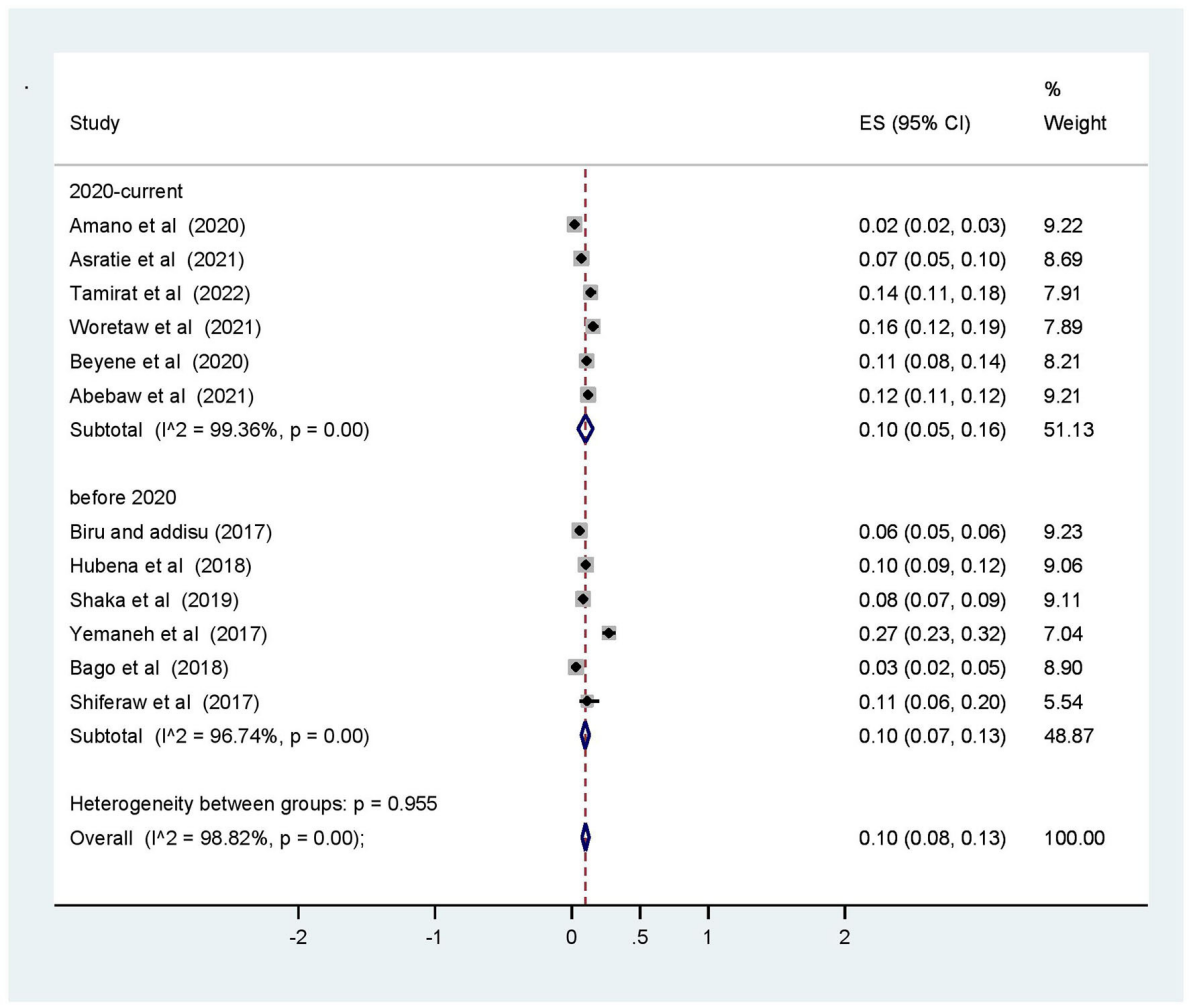
Subgroup analysis based on year of publication for operative vaginal deliveries among mothers who gave birth in Ethiopia, 2022.

**Figure 6:**
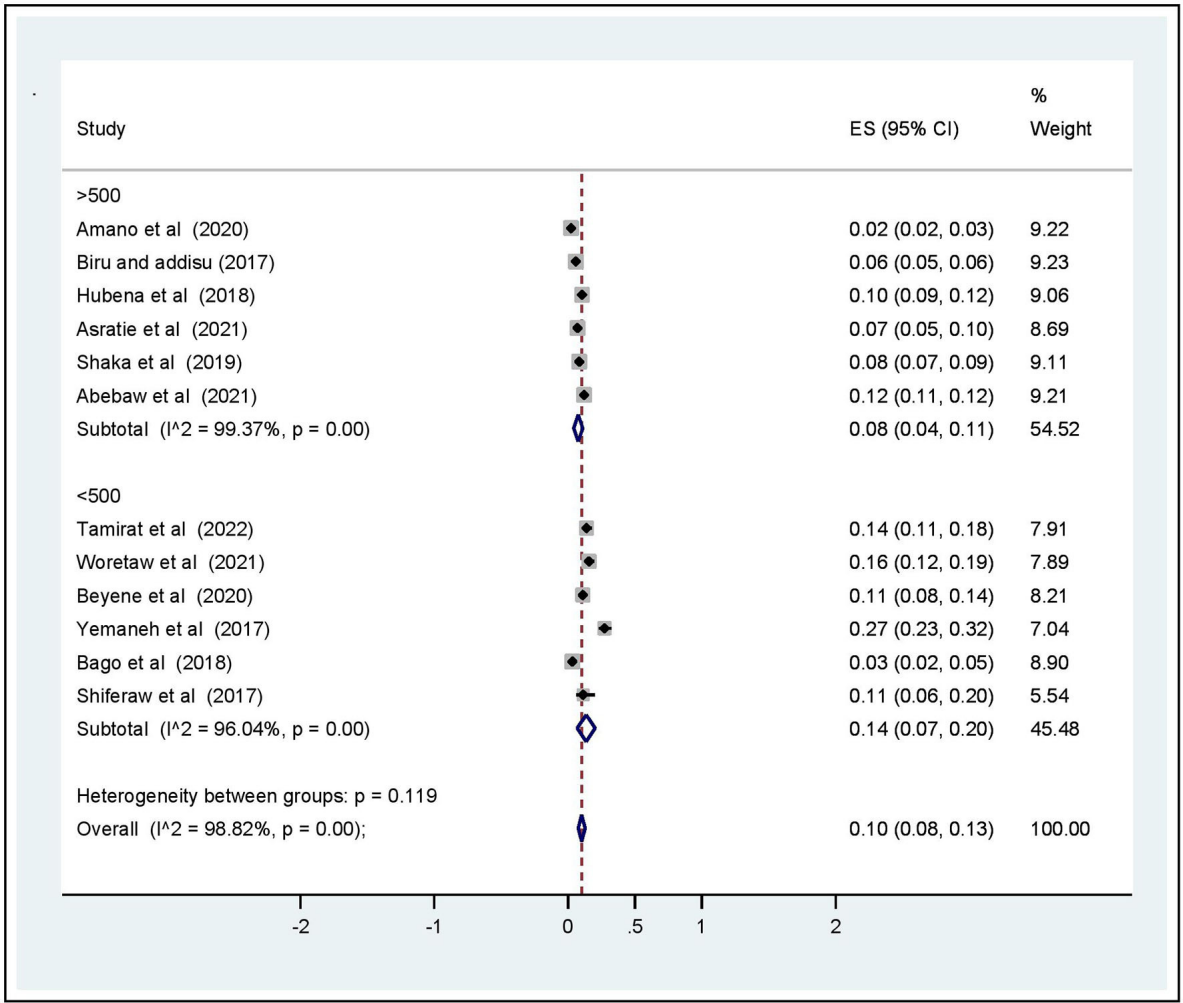
Subgroup analysis based on sample size for prevalence of operative vaginal deliveries among mothers who gave birth in Ethiopia, 2022.

Subgroup analysis was computed with the evidence of heterogeneity. Hence the Cochrane I^2^ statistic = 98.82%, *P* < 0.001) showed the presence of marked heterogeneity in this study. Therefore, subgroup analysis was implemented using the study area (region), year of publications, and sample size using random model effect analysis. Regarding the study area (region), the prevalence of OVDs was highest in Addis Ababa accounting for 27% (95% CI: 11, 12) whereas the rate of OVDs was higher among studies having a sample size of <500, accounting for 14% (95% CI: 7–20) (Figures 4–6).

### Leave-out-one sensitivity analysis

Leave-out-one analysis was done to evaluate the effect of each study on the pooled estimated prevalence of operative vaginal delivery by excluding each study step by step. The result showed that the excluded studies did not show a significant effect on the estimated prevalence of operative vaginal delivery among mother who gave birth in Ethiopia (Table 3).

**Table 3:** Sensetivity analysis on studies included for the systematic review and meta-analysis on OVDs among mothers who gave birth in Ethiopia 2022.

**Study left out during sensitivity analysis**	**Pooled prevalence of OVD(95% CI)**	***P*-value**
Biru et al. (13)	10 (8,11)	0.00
Hubena et al. (19)	9 (5,13)	0.00
Abebaw et al. (20)	10 (8,11)	0.00
Shaka et al. (14)	10 (8,11)	0.00
Asratie et al. (21)	9 (5,13)	0.00
Tamirat et al. (22)	10 (8,11)	0.00
Beyene et al. (24)	9 (5,13)	0.00
Bago et al. (25)	9 (5,13)	0.00
Shiferaw et al. (26)	9 (5,13)	0.00
Yemaneh et al. (7)	10 (8,11)	0.00
Abdo et al. (6)	10 (8,11)	0.00
Woretaw et al. (23)	10 (8,11)	0.00

### Feto-maternal indications for OVDs in Ethiopia

Fetal distress, prolonged labor, and maternal exhaustion were the feto-maternal indications of OVDs in Ethiopia with a pooled prevalence of 40%, 27%, and 23%, respectively (Table 4).

**Table 4:** Feto-Maternal indications for operative vaginal deliveries among women who gave births in Ethiopia, 2022.

**Feto-maternal indications**	**Model**	**Status of heterogeneity**	**Prevalence (95%CI)**	***I*^2^ (%)**	***P*-value**
Fetal distress (14, 30, 32, 38–41)	Random	Marked	40 (34–47)	91.18	≤0.001
Prolonged labor (14, 30, 32, 38, 39, 41)	Random	Marked	27 (22–32)	84.92	≤0.001
Maternal exhaustion (13, 14, 26, 40)	Random	Marked	23 (12–34)	92.75	≤0.001

### Feto-maternal outcomes following OVDs in Ethiopia

Operative vaginal deliveries can cause significant complications, disability, or death, particularly in settings that lack the skillful health professionals who have no the capacity to properly conduct safe forceps and/or vacuum-assisted delivery per the prerequisites. Likewise, neonatal death, poor APGAR score, admission to NICU, perianal tear, and postpartum hemorrhage were the feto-maternal complications following the OVDs in Ethiopia with a pooled prevalence of 18, 11, 25, 2, and 3%, respectively (Table 5).

**Table 5:** Feto-Maternal complications following OVDs among women who gave births by OVD in Ethiopia, 2022.

**Feto-maternal complications**	**Model**	**Status of heterogeneity**	**Prevalence (95%CI)**	***I*^2^ (%)**	***P*-value**
Admitted to NICU (14, 39)	Random	Less	18 (14–21)	0.00	0.1
Perianal tears (14, 30, 40, 41)	Random	Marked	11 (2–20)	97.20	≤0.001
Poor APGAR score (14, 37, 39, 40)	Random	Marked	25 (4–47)	99.17	≤0.001
Neonatal death (37, 39)	Random	Less	2 (0–3)	0.0	0.1
PPH (14, 39, 41)	Random	Marked	3 (0–5)	78.78	0.01

NICU, newborn intensive care unit; APGAR, Appearance, Pulse, Grimace, Activity, and Respiration; PPH, Post-Partum Hemorrhage.

## Discussion

This systematic review and meta-analysis showed summarized prevalence of OVDs, its indications, and outcomes among mothers who gave birth in Ethiopia. The proportion of OVDs is found to be high in the country.

The finding from this systematic review and meta-analysis showed the overall pooled prevalence of OVDs among mothers who gave birth in Ethiopia was 10% (95% CI: 8–13). This implies 10 out of a hundred women giving birth undergo operative vaginal deliveries to shorten the second stage of labor for fetal and maternal indications. This is probably because OVDs have less propensity to resort than cesarean-section, less pre-and postnatal blood loss, and less need for analgesia. In addition, the procedures are easy to learn compared to that of caesarian-section (27–29). The finding from this review conforms with the finding from the United kingdom and recommendation of Royal College of obstetrics and gynecology instrumental vagina delivery (30).

However, this report is higher than the studies done in India (18), Nepal (31), and Nigeria (32–34). In addition, the finding from this review was high compared to a survey conducted by WHO in nine Asian countries which found a prevalence of 3.2% (35). This discrepancy might be due to the variability in the number of the studies involved in the analysis since the current finding is an aggregate of many studies while the finding from listed countries was from single studies with high sample size. Moreover, the variation in the accessibility of skilled health professionals who might conduct the procedure also contributes to the discrepancy.

In this review operative, vaginal deliveries were indicated for fetal distress, prolonged labor, and maternal exhaustion for 40, 27, and 23% respectively. This is because most of the time OVDs are indicated to shorten the second stage of labor. Similarly, a study done in Nigeria revealed as fetal distress, prolonged labor, and maternal exhaustion are the major indications of OVDs (34, 36).

Furthermore, the finding from this review indicated that OVDs contributes to both maternal and neonatal complications. Accordingly, this review revealed that OVDs contribute to feto-maternal complications like NICU admission, perianal tear, neonatal death, and PPH for 18, 11, 2, and 3%, respectively. Even though OVDs aimed to save maternal and neonatal life with minimal complications, feto-maternal complications in this systematic review and meta-analysis are alarming and severe. The finding from this review was consistent with the finding from other developing countries. This might be linked to a less equipped setup, scarce skillful human power, and many health professionals do not consider prerequisites of OVDs (1, 37).

## Strengths and limitations

The investigators used extensive and comprehensive search strategies from multiple databases. Published as well as unpublished studies and gray literature were included. Studies were evaluated for methodological quality using a standardized tool. Although the literature search was systematic and assessed all related studies within the desired scope, some relevant publications, for instance, publications reported in non-English language and local languages, may have been missed.

## Conclusion

This systematic review and meta-analysis revealed that one in a 10 mothers who gave birth undergo OVDs in Ethiopia. Neonatal death, poor Apgar score, admission to NICU, perianal tear, and PPH were the most common feto-maternal complications following the OVDs. Avoiding unjustified and unnecessary indications for OVDs has a significant impact to prevent poor feto-maternal outcomes. Thus, concerned stakeholders should encourage quality OVDs practice by avoiding unnecessary indications and scaling up the skill of health professionals who might conduct OVDs through special training.

## Data availability statement

The original contributions presented in the study are included in the article/Supplementary material, further inquiries can be directed to the corresponding authors.

## Author contributions

BB, BE, HB, EZ, AA, and AD conceived and designed the review. BB and BE developed the search strings. The two reviewers (BB and BE) screened and selected studies. BB, BE, HB, EZ, IK, and ADW carried out the draft of the manuscript, and BB is the PI of the review. Likewise, all authors extracted the data and evaluated the quality of the studies. BB, HB, IK, and SH carried out the analysis and interpretation. BB, BE, HB, EZ, SH, AA, and AD rigorously reviewed the manuscript. All authors read and approved the final version of the manuscript.

## Conflict of interest

The authors declare that the research was conducted in the absence of any commercial or financial relationships that could be construed as a potential conflict of interest.

## Publisher's note

All claims expressed in this article are solely those of the authors and do not necessarily represent those of their affiliated organizations, or those of the publisher, the editors and the reviewers. Any product that may be evaluated in this article, or claim that may be made by its manufacturer, is not guaranteed or endorsed by the publisher.
