# Supplementary figures and images for: Prevalence, indications, and outcomes of operative vaginal deliveries among mothers who gave birth in Ethiopia: A systematic review and meta-analysis
Source: Front Glob Womens Health. 2022 Sep 22;3:948288. doi: 10.3389/fgwh.2022.948288 (PMC9535624; doi:10.3389/fgwh.2022.948288)

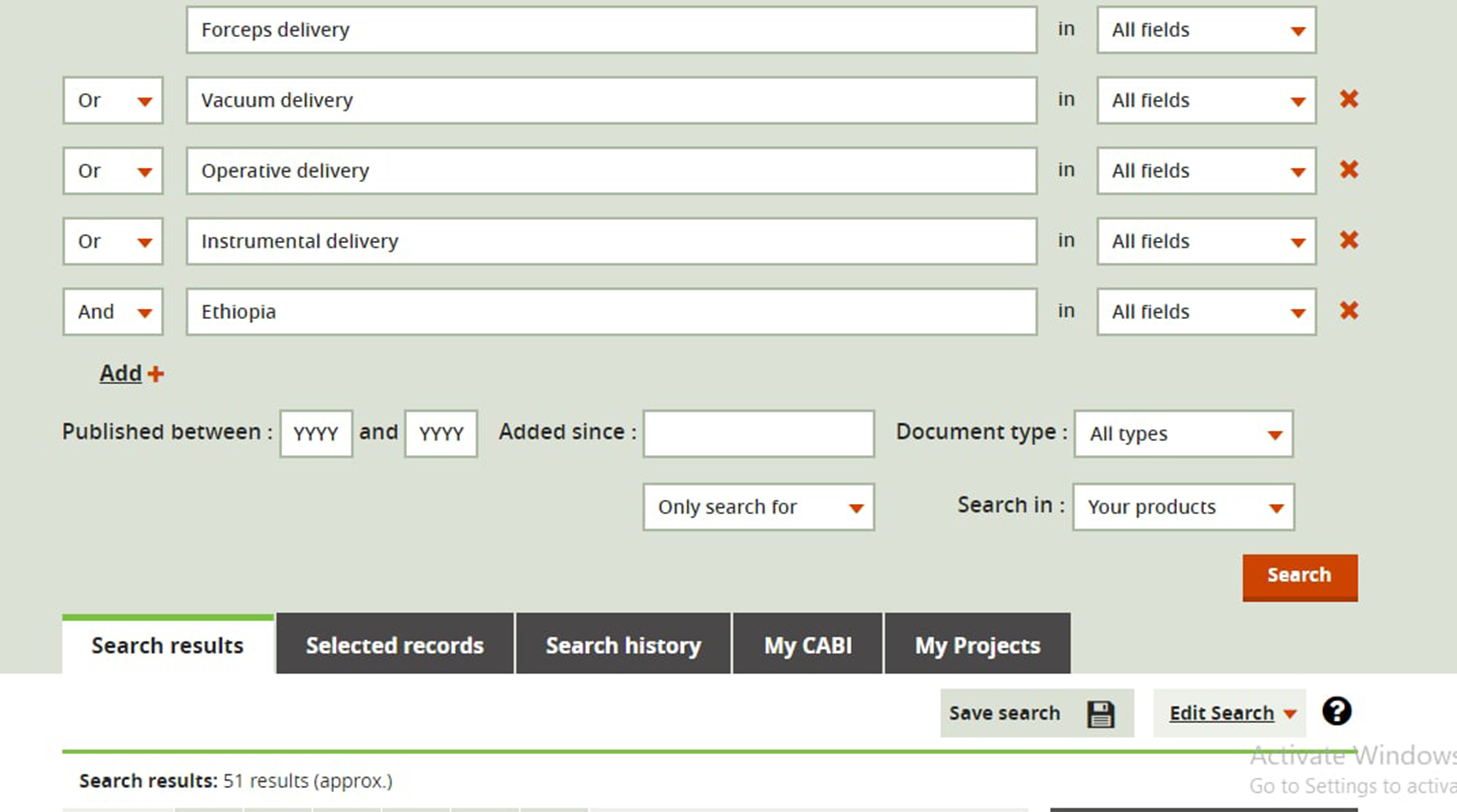

Supplement: Supplementary file 1 [file Image_1.JPEG]

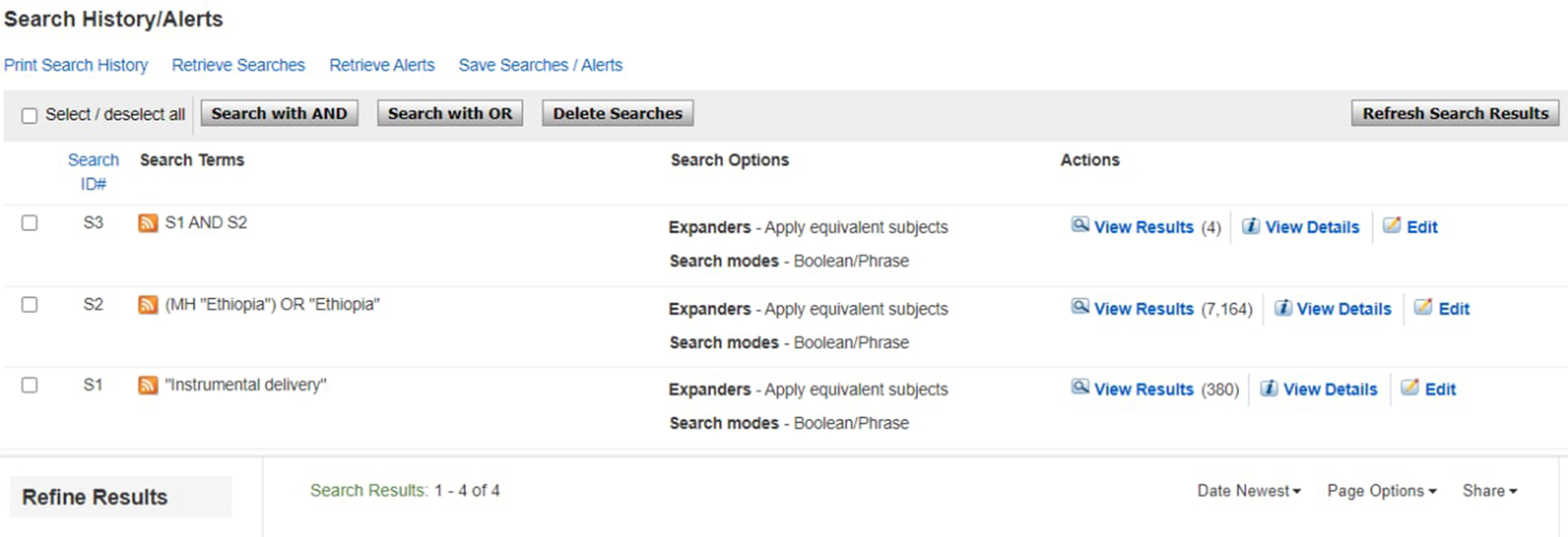

Supplement: Supplementary file 2 [file Image_2.JPEG]

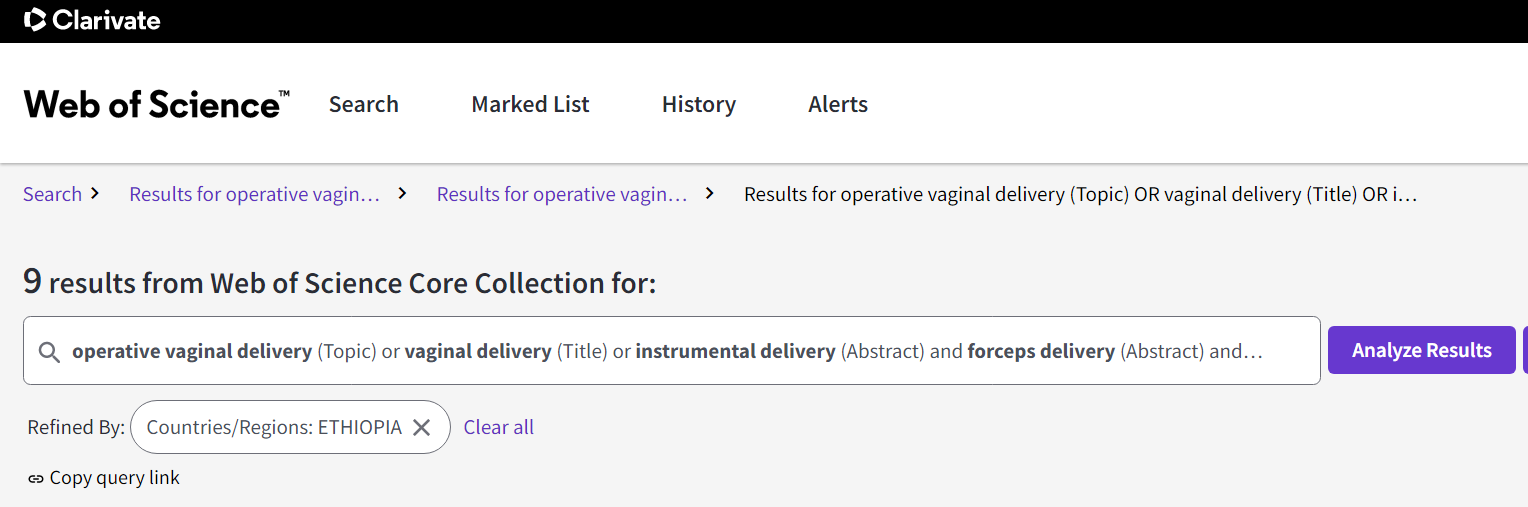


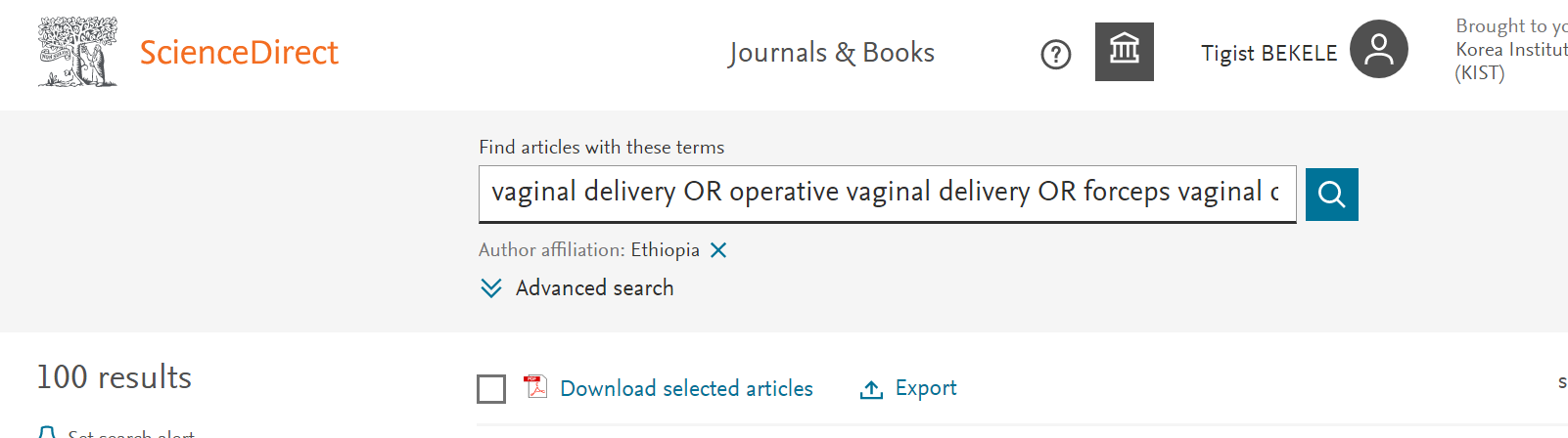


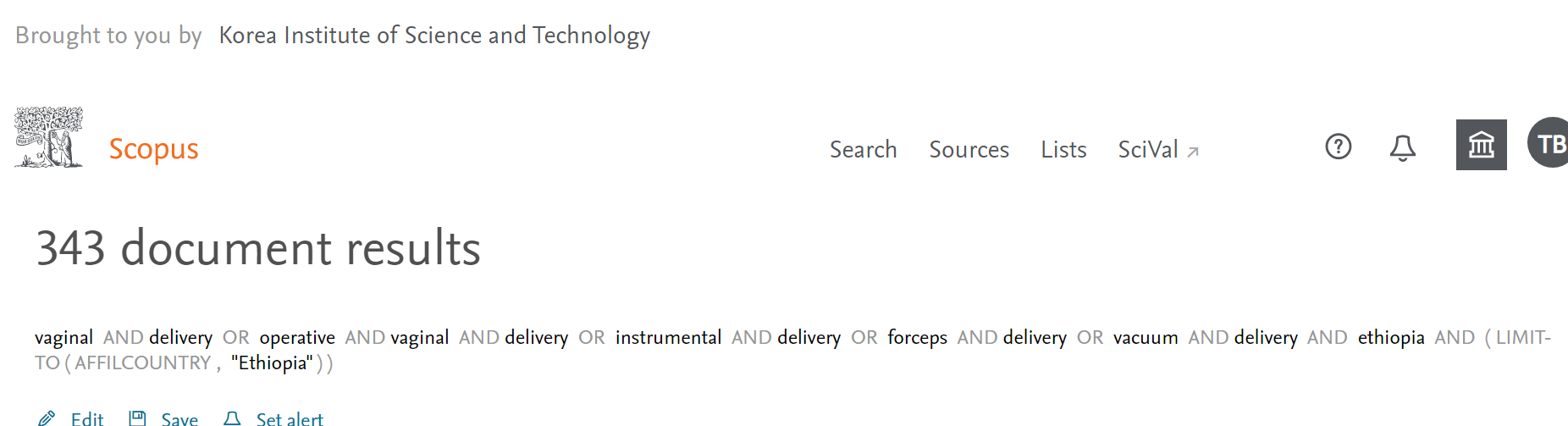

Supplement: Supplementary file 4 [file Table_2.docx]
