# Supplementary material for: Prevalence, indications, and outcomes of operative vaginal deliveries among mothers who gave birth in Ethiopia: A systematic review and meta-analysis
Source: Front Glob Womens Health. 2022 Sep 22;3:948288. doi: 10.3389/fgwh.2022.948288 (PMC9535624; doi:10.3389/fgwh.2022.948288)
Supplement: Supplementary file 3 [file Table_1.docx]

**Appraisal**

Table 1: Critical appraisal check list of quantitative studies of Operative vaginal deliveries, indications, and outcomes among mothers who gave birth in Ethiopia (1 =yes, 0=no/not mentioned); total score=8

| Studies | Q1 | Q2 | Q3 | Q4 | Q5 | Q6 | Q7 | Q8 | Total score |
| --- | --- | --- | --- | --- | --- | --- | --- | --- | --- |
| Amano et al | Y | Y | Y | N | Y | Y | Y | Y | 7 |
| Biru and addisu | Y | Y | Y | Y | Y | N | Y | Y | 7 |
| Hubena et al | Y | Y | Y | Y | Y | Y | Y | Y | 8 |
| Asratie et al | Y | Y | Y | Y | Y | Y | Y | Y | 8 |
| Tamirat et al | Y | Y | Y | N | N | Y | Y | Y | 6 |
| Shaka et al | Y | Y | Y | N | Y | Y | Y | Y | 8 |
| Woretaw et al | Y | Y | Y | Y | Y | Y | Y | Y | 8 |
| Beyene et al | Y | Y | Y | N | Y | Y | Y | Y | 7 |
| Yemaneh et al | Y | Y | Y | N | Y | Y | Y | Y | 7 |
| Bago et al | Y | Y | Y | Y | Y | Y | Y | Y | 8 |
| Shiferaw et al | Y | Y | N | Y | Y | Y | Y | Y | 7 |
| Abebaw et al | Y | Y | Y | Y | Y | Y | Y | Y | 8 |

**Notes:**

Q1 - Were the criteria for inclusion in the sample clearly defined?

Q2 - Were the study subjects and the setting described in detail?

Q3 - Was the exposure measured in a valid and reliable way?

Q4 - Were objective, standard criteria used for measurement of the condition?

Q5 - Were confounding factors identified?

Q6 - Were strategies to deal with confounding factors stated?

Q7 - Were the outcomes measured in a valid and reliable way?

Q8 - Was appropriate statistical analysis used?

Abbreviations: Y, yes; N, no/ Not mentioned; U, unclear
